# Underwater compression hemostasis method for active bleeding in underwater endoscopic submucosal dissection

**DOI:** 10.1055/a-2619-1271

**Published:** 2025-07-29

**Authors:** Mitsuru Nagata

**Affiliations:** 1 370772 Department of Endoscopy, Shonan Fujisawa Tokushukai Hospital, Fujiwasa, Japan

Active bleeding during underwater endoscopic submucosal dissection (U-ESD) in saline can cause visual field loss 1 . The field can be visualized by changing to under gas conditions, and hemostasis can be achieved using a knife or hemostatic forceps. However, this approach may cause a large amount of blood to mix with the saline, and deaeration and saline replacement are required before the resumption of U-ESD. Moreover, if active bleeding occurs on the gravity side where U-ESD can be suitable 2 , blood accumulation due to gravity makes identifying the bleeding point difficult even under gas conditions, missing appropriate timing for hemostasis.

To address these issues, we report the underwater compression hemostasis method ( Fig. 1 ). In this method, when bleeding causes visual field loss, the area suspected of bleeding is immediately compressed with the hood tip while applying the water jet. If compression hemostasis is successful and the visual field clears, the compression can be slightly released to confirm the bleeding point, followed by hemostasis using a knife or hemostatic forceps. If the bleeding point is distant from the accessory channel, the accessory channel should be moved gradually closer to the bleeding point while maintaining compression and applying the water jet. Finally, hemostasis using a knife or hemostatic forceps can be performed. If bleeding deep in the dissection plane occurs, the bleeding point cannot be compressed ( Fig. 2 ). Therefore, blind dissections deep in the dissection plane should be avoided to successfully manage bleeding during U-ESD.

**Fig. 1:**
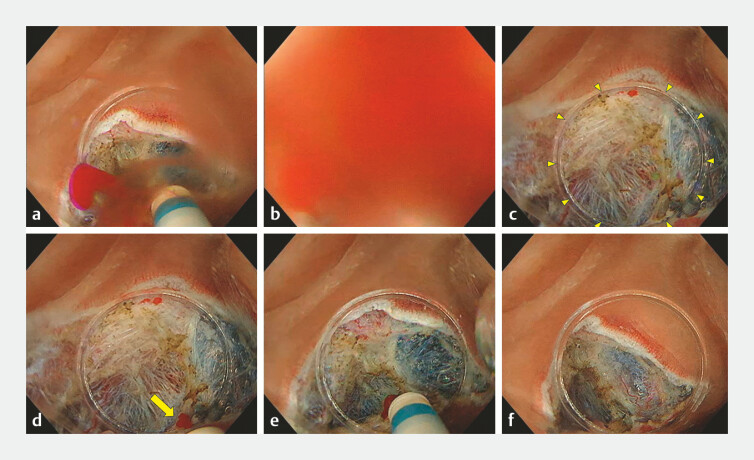
Underwater compression hemostasis method during underwater endoscopic submucosal dissection. **a** Active bleeding occurred on the gravity side during submucosal dissection. **b** The visual field was immediately lost due to active bleeding. **c** The hood tip (yellow arrowheads) was compressed on the area suspected of bleeding. **d** The hood tip was moved slightly to confirm the bleeding point (yellow arrow). **e** Hemostasis was achieved using the DualKnife J (Olympus, Tokyo, Japan) and the preciseSECT (Effect 3.0) of the VIO3 (Erbe, Tübingen, Germany) in saline while compressing the bleeding point. **f** The compression was released and successful hemostasis was confirmed.

**Fig. 2:**
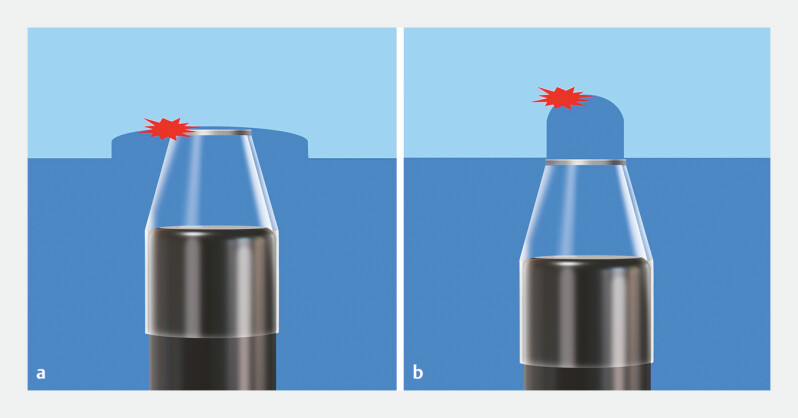
Exposed bleeding points and bleeding points deep in the dissection plane during underwater endoscopic submucosal dissection. **a** The exposed bleeding point can be compressed with the hood tip. **b** The bleeding point deep in the dissection plane cannot be compressed with the hood tip.

Video 1 shows the underwater compression hemostasis method for active bleeding on the gravity side using a hood with self-made air bubble outlets 3 4 5 . This method could be performed despite the smaller area of the hood. In conclusion, the underwater compression hemostasis method can be employed to manage active bleeding in U-ESD, particularly on the gravity side.

Underwater compression hemostasis method for active bleeding during underwater endoscopic submucosal dissection.Video 1

Endoscopy_UCTN_Code_TTT_1AQ_2AF

Citation Format

Endoscopy 2025; 57: E257–E258. DOI: 10.1055/a-2556-5656

## Notes

Endoscopy E-Videos https://eref.thieme.de/e-videos
*E-Videos* is an open access online section of the journal *Endoscopy* , reporting on interesting cases and new techniques in gastroenterological endoscopy. All papers include a high-quality video and are published with a Creative Commons CC-BY license. Endoscopy E-Videos qualify for HINARI discounts and waivers and eligibility is automatically checked during the submission process. We grant 100% waivers to articles whose corresponding authors are based in Group A countries and 50% waivers to those who are based in Group B countries as classified by Research4Life (see: https://www.research4life.org/access/eligibility/ ). This section has its own submission website at https://mc.manuscriptcentral.com/e-videos .
